# Role of cineole in alleviation of acute kidney injury and renal function recovery following gentamicin administration in rats

**DOI:** 10.22038/IJBMS.2023.68430.14944

**Published:** 2023

**Authors:** Sobhan Rahimi Monfared, Ali Valibeik, Leila Jafaripour, Zahra Eslamifar, Saeed Veiskarami, Hassan Ahmadvand

**Affiliations:** 1 Razi Herbal Medicines Research Center, Lorestan University of Medical Sciences, Khorramabad, Iran; 2 Student Research Committee, Lorestan University of Medical Sciences, Khorramabad, Iran; 3 Department of Clinical Biochemistry, School of Medicine, Lorestan University of Medical Sciences, Khorramabad, Iran; 4 Department of Anatomy, School of Medicine, Dezful University of Medical Sciences, Dezful, Iran; 5 Department of Medical Laboratory Sciences, School of Paramedical Sciences, Dezful University of Medical Sciences, Dezful, Iran; 6 Lorestan Agricultural and Natural Resources Research and Education Center, Department of Animal Science, Khorramabad, Iran; 7 Medical Plants and Natural Products Research Center, Hamadan University of Medical Sciences, Hamadan, Iran

**Keywords:** Cineole, Gentamicin, Nephrotoxicity, Oxidative stress, Rat

## Abstract

**Objective(s):**

Gentamicin leads to kidney failure by producing free radicals and inflammation in renal tissue. Cineole as a terpenoid has antioxidant properties. Antioxidants can play an effective role in preserving the oxidant-antioxidant balance. Hence, this study investigated the effects of cineole on acute kidney injury (AKI) and renal function recovery following gentamicin administration in rats.

**Materials and Methods:**

36 male Wistar rats were randomly divided into 6 equal groups; healthy control, gentamicin, DMSO carriers, cineole 50, cineole 100, and vitamin E. After 12 days of treatment, the animals were anesthetized with ketamine and xylazine. Serum and kidney samples were taken for biochemical and gene expression experiments.

**Results:**

Cineole 50 and 100 groups increased the levels of serum glutathione (GSH) (<0.05), kidney and serum glutathione peroxidase (GPX) (<0.001), kidney catalase (CAT) (<0.001), serum nitric oxide (NO) (<0.001), and the GPX gene (<0.05) compared with the gentamicin group. These treatment groups had decreased levels of kidney malondialdehyde (MDA) (<0.001), serum creatinine (<0.001), urine protein, and the Interleukin 6 (IL-6) gene (<0.05) compared with the gentamicin group. Cineole 50 increased the serum MDA (<0.001), urea, and CAT gene (>0.05) and decreased the kidney GSH (<0.05) and the tumor necrosis factor-alpha (TNF-α) gene (<0.05). Cineole 100 increased the kidney GSH (<0.05) and decreased the serum MDA (<0.001), urea, CAT gene (>0.05), and TNF-α gene (>0.05) compared with the gentamicin group. Improvement in histological alterations was displayed in cineole groups compared with the gentamicin group.

**Conclusion:**

Cineole can reduce kidney damage caused by nephrotoxicity following gentamicin consumption through its antioxidant and anti-inflammatory properties.

## Introduction

Renal ischemia and toxic agents cause renal failure, structural damage, and decreased renal function, which are the characteristics of acute kidney injury (AKI) (1). Nephrotoxic medicinal agents such as cisplatin and gentamicin cause kidney tissue inflammation and damage to the proximal renal tubules (2, 3). Gentamicin is an aminoglycoside antibiotic widely used in the treatment of gram-negative bacterial infections, however, its use is limited by serious side effects such as nephrotoxicity (4). Previous studies have demonstrated that this aminoglycoside antibiotic is significantly capable of causing nephrotoxicity in humans and laboratory animals (4). 

Induction of oxidative stress through free radicals causes nephrotoxicity following gentamicin administration. The reduction of antioxidant capacity and lipid peroxidation in the proximal convoluted renal tubule is caused by free radicals (5, 6). Production of reactive oxygen species (ROS) and reactive nitrogen species (RNS), activation of inflammatory processes, reduction in antioxidant defense, and contraction of mesenchymal cells with reduced renal blood flow, lead to tubular necrosis and leukocyte infiltration. Finally, they decrease the glomerular filtration rate (GFR) and impair renal function (7, 8). Gentamicin-induced nephrotoxicity increases pro-inflammatory cytokines such as tumor necrosis factor-alpha (TNF-α) and interleukin-6 (IL-6) (9). 

Today, evidence suggests that the usage of antioxidants can increase the body’s antioxidant enzymatic system, especially catalase (CAT) and superoxide dismutase (SOD), and inhibit or decrease the damaging effects of reactive oxygen species (10). L-glutamine, camphor, and pomegranate with their anti-inflammatory and antioxidant properties can reduce nephrotoxicity induced by gentamicin (11, 12). 

Cineole is also known as eucalyptol and cajeputol. This compound is found as an oxidized monoterpene in eucalyptus (75%), *Rosmarinus Officinalis *(40%), and *Myrtus Communis *(37%). Studies have shown that cineole has anti-inflammatory and anti-coagulant effects, furthermore, oral administration of cineole in rats reduces edema (13). Cineole reduces glutathione by decreasing myeloperoxidase activity (14) and, in addition to lowering blood sugar, is effective in increasing glucose uptake from muscles by enhancing insulin secretion (15). Cineole usage in the diet is recommended to prevent intestinal and gastric ulcers (14), moreover, it reduces aortic pressure and heart rate (16).

Since gentamicin has many applications as an antibiotic and kidney damage following its usage is inevitable, administration of natural antioxidant compounds has several benefits. Hence, the purpose of our study was to evaluate the antioxidant effects of cineole on alleviation of renal tissue damage following gentamicin injection in rats by investigating kidney function tests, antioxidant enzymes, and tissue parameters.

## Materials and Methods

***Chemicals***

Tris-ethylenediaminetetraacetic acid (Tris-EDTA) and Tris-HCl were provided by the Merck Company (Germany). 2,4-Dinitro thiocyanate benzene (DNTB), NaN_3_, gentamicin, hematoxylin, eosin, cineole, and vitamin E were purchased from Sigma-Aldrich Company (USA). Commercial kits for the measurement of urea and Cr were taken from Pars Azmoon Company (Iran). A complementary DNA (cDNA) synthesis kit and SYBER Green qPCR Master Mix 2x were purchased from Yekta Tajhiz Azma (YTA) Company (Iran).

***Laboratory animals***

All animal experiments in the present study are approved by the ethics committee of Lorestan University of Medical Sciences, Iran (IR.lums.REC.1396.358), based on internationally accepted guidelines for the usage of animals. 

During this experimental research, thirty-six male Wistar rats (in the age range of 10 to 12 weeks with a mean weight of 180±30 g) were used. These animals were kept at a sustained room temperature of 23±2 ^°^C, humidity of 50-55 %, and 12 hr light/dark cycle. They had free access to water and food during the experimental period. After two weeks of adapting to the environmental conditions, the animals were randomly divided into 6 equal groups and all injections were performed intraperitoneally. 

***Experimental groups***

1. Healthy control group: Daily injection of saline equal to the volume of experimental groups for 12 days

2. Gentamicin group: Daily injection of gentamicin (100 mg/kg) and saline for 12 days (17). 

3. DMSO carrier group: Daily injection of gentamicin (100 mg/kg) and DMSO for 12 days.

4. Cineole 50 (Treatment 1) group: Daily injection of gentamicin (100 mg/kg) and treatment with cineole at a dose of 50 mg/kg for 12 days (18). 

5. Cineole 100 (Treatment 2) group: Daily injection of gentamicin (100 mg/kg) and treatment with cineole at a dose of 100 mg/kg for 12 days (18). 

6. Vitamin E (Positive Control) group: Daily injection of gentamicin (100 mg/kg) and treatment with vitamin E (1 mg/g) for 12 days (19).

cineole was dissolved in DMSO (20). Saline, DMSO, cineole, and vitamin E were intraperitoneally injected one hour after gentamicin. 

After twelve days, 50 mg/kg ketamine and 5 mg/kg xylazine were utilized to anesthetize the animals** (**21**).** The blood samples were taken from the animal’s hearts, the abdomen of the animals was shaved and a midline laparotomy was created. The intestines were pushed aside to expose the kidneys. The kidneys were divided into two equal sections, then a section of the tissue was fixed in 10% buffered formaldehyde solution for histopathological studies and for biochemical parameters and gene expression, kept at -80 ^°^C freezer.

***Serum analysis***

The blood sample was centrifuged at 3000 rmp to separate the serum. Serum was stored in the freezer at -80 ^°^C for the next steps to measure the serum factors in this study, including glutathione peroxidase (GPX), CAT, glutathione (GSH), malondialdehyde (MDA), nitric oxide (NO), urea, and creatinine.

***Measurement of biochemical parameters***

To evaluate renal biochemical parameters, For each gram of kidney tissue, 10 ml of PBS buffer was added, then it was centrifuged at 15000 rpm at 4 ^°^C for 15 min. The supernatant was isolated and stored in microtubes at -80 ^°^C. Isolated tissue extracts were used to measure the level or activity of tissue factors including GPX, CAT, GSH, and MDA. 

The amount of protein in tissue extracts was measured to determine the concentration of antioxidant enzymes using the Bradford method (22). 180 μl of the Bradford solution was poured into a tube, and 20 μl of serum or supernatant sample of homogenized tissue was added. The absorption of the samples was read at 595 nm by a spectrophotometer. Urine protein was measured by the turbidity method, for this purpose, trichloroacetic acid (TCA) 3% was added then the absorption of the samples was read at 420 nm by a spectrophotometer instrument.

CAT was measured by the Aebi method (23) with a slight change. Briefly, a mixture of Potassium phosphate buffer (PH=7), peroxide hydrogen, and kidney sample was prepared and then its absorption was read using a spectrophotometer at 240 nm in 0, 30, and 60 sec. 

GPX was measured according to literature (24) with a slight change. This assay was performed using Tris-Hcl buffer, sodium azide, a homogeneous tissue serum sample, glutathione, TCA, Tris- EDTA, DNTB, and hydrogen peroxide. Then, the absorption was read at 420 nm using an ELISA reader (AWARENESS STAT FAX-2100, America).

For GSH, a mixture of Tris- EDTA buffer (pH=8), DTNB (5,5′-dithiobis-2-nitrobenzoic acid), serum, or homogenized tissue sample was prepared and the ELISA reader (AWARENESS STAT FAX-2100, America) read its absorbance at 412 nm. 

To evaluate MDA, a mixture of Thiobarbituric acid (TBA), TCA, and serum sample, or homogenized tissue supernatant was used, and then, the spectrophotometer instrument read its absorbance at 535 nm. 

To measure NO, a Griss reagent and a serum sample were used. The absorption was read at 560 nm using an ELISA reader (25). Serum urea and creatinine were measured using a Pars Azmon test kit.

***Molecular analysis (Real-time PCR)***

Kidney tissue was used to measure the expression of CAT, GPX, TNF-α, and IL-6 genes. For this purpose, RNA extraction was carried out based on Rio *et al*. with a slight change (26). The conversion of RNA into cDNA was carried out using a commercially available kit (YTA, Yekta Tajhiz Azma, Iran) based on the manufacturer’s instructions. Multiplex real-time polymerase chain reactions (PCR) were performed. The reaction mixture included 2x SYBR Green qPCR Mix (1 X), 0.2 µl of primers for the favorable genes (Table 1), 1 µl of cDNA template, and 8.2 µl of RNAase-free water (DEPC water). The final volume of the reaction mixture (20 µl) was evaluated for proliferation under specified conditions: Initial denaturation step at 95 ^°^C for 3 min; then 40 cycles of denaturation for 5 secat 95 ^°^C, and annealing for 30 sec at 60 ^°^C, followed by 5 min final extension step at 50-99 ^°^C using a Real-Time PCR System thermocycler. The β-actin gene was used as a housekeeping gene that is expressed in all cells.

***Histological studies***

The rats’ kidneys were placed in a 10% buffered formaldehyde solution after separation. After dehydration with alcohol in ascending grades and clarification with xylene, the blocks were prepared. Then, a 5-micrometer section was prepared with a microtome. Hematoxylin and eosin were used for tissue staining. Tissue sections were examined for histological changes by a light microscope with 400× magnification. Then tubular necrosis, eosinophilic casts, leukocyte infiltration, and atrophic glomeruli were evaluated semi-qualitatively by the method of Caramello *et al.* (27). These injuries were scored as follows: No damage=0, mild damage (one cell damage)=1, moderate damage (damage less than 25%)=2, severe damage (damage between 25-50%)=3, and very severe damage (damage more than 50%)=4. 

***Statistical analysis***

Data analysis was performed using SPSS version 16.0 statistical software. The normal distribution of the data was checked using the Shapiro-Wilk test. One-way analysis of variance (ANOVA) and Tukey’s post-tests were utilized for comparisons. The values were represented by mean±standard deviation (SD). In all tests, the probability level was *P*<0.05. The method utilized to analyze the expression of genes was based on the delta-delta Ct (∆∆Ct) method. The GraphPad Prism 8 software package is used to display the graphs.

## Results

***Effect of cineole on biochemical parameters in the urine, serum, and extracted renal tissue***

**Figure 1:**
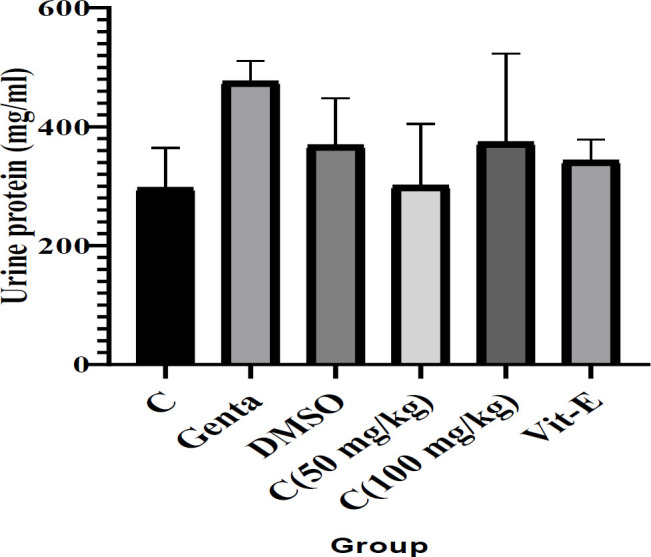
Urine protein levels in experimental groups following gentamicin-induced nephrotoxicity and treatment with cineole and vitamin E in rats (n=6). Data were expressed as mean±SD. Data were analyzed by one-way ANOVA followed by Tukey’s *post hoc* test

Table 2 shows the results of the analysis of the activity of antioxidant enzymes (CAT, GSH, and GPX), serum NO, serum creatinine, and MDA of serum and kidney tissue in experimental groups following gentamicin injection and cineole treatment. Furthermore, the results of the analysis of the serum urea and urine protein are shown in Table 3 and Figure 1, respectively. 

Tables and figures show that in general, DMSO as a carrier did not have a significant effect on the investigated parameters compared with the control group. The results demonstrated that gentamicin resulted in a remarkable decrease in kidney CAT (<0.001) and serum MDA (<0.001) activity levels and a significant increase in serum urea and creatinine levels (<0.001) compared with the healthy control group. Moreover, gentamicin increased the kidney MDA and serum NO levels (<0.001) and decreased the kidney and serum GSH (<0.05) and GPX (<0.001) levels. 

Cineole 50 and 100 groups increased the levels of serum GSH (<0.05), kidney and serum GPX (<0.001), kidney CAT (<0.001), and serum NO (<0.001) compared with the gentamicin group, although the difference was not significant in some parameters. Moreover, these treatment groups had decreased levels of kidney MDA (<0.001), and serum creatinine (<0.001) compared with the gentamicin group. Cineole 50 had increased serum MDA (<0.001) and serum urea and decreased kidney GSH, although the difference was not significant in these parameters. Cineole 100 had increased serum and kidney GSH and deceased kidney MDA (<0.001) and serum urea compared with the gentamicin group, although the difference was not significant in some parameters. 

Vitamin E caused an increase in serum and kidney GSH compared with the gentamicin group, although this increase was not significant. Also, vitamin E caused an increase in serum and kidney GPX (<0.001), and kidney CAT (<0.001) activities compared with the gentamicin group. On the other hand, it significantly decreased the kidney MDA (<0.001) and serum creatinine levels compared with the gentamicin group.

***Effect of cineole on gene expression of antioxidant enzymes and inflammatory factors***

**Figure 2:**
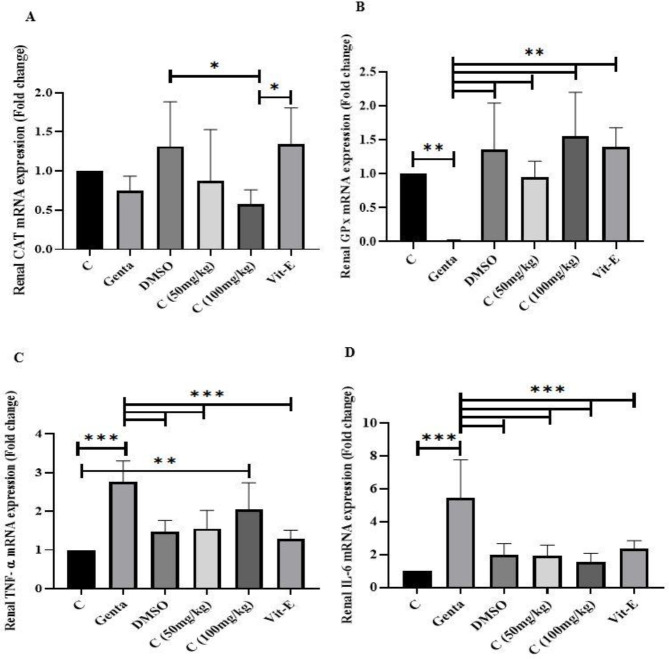
Gene expression of CAT, GPX, TNF-α, and IL-6 following gentamicin-induced nephrotoxicity and treatment with cineole and vitamin E in rats (n=6). Data were expressed as mean±SD. Data were analyzed by one-way ANOVA followed by Tukey’s post hoc test

The results of the effects of cineole and vitamin E on gene expression are shown in Figure 2. 

Gentamicin caused an increase in TNF-α (*P*<0.05) and IL-6 (*P*<0.05) gene expression and a decrease in CAT (*P*>0.05) and GPX (*P*<0.05) genes compared with the control group, although in CAT genes expression decrease was not significant. The cineole 50 treatment group increased the CAT gene expression (*P*>0.05), however, cineole 100 decreased this gene (*P*>0.05) compared with the gentamicin group, although these differences were not significant. The expression of the GPX gene increased in both treatment groups (*P*<0.05) and the expression of the IL-6 gene decreased significantly in cineole groups compared with the gentamicin group (*P*<0.05). The TNF-α gene expression decreased in both treatment groups compared with the gentamicin group, as this decrease was significant in the cineol 50 group (*P*<0.05) and insignificant in the cineol 100 group (*P*>0.05) (Figure 2). 

Moreover, vitamin E increased the expression of CAT (*P*>0.05) and GPX (*P*<0.05) genes and decreased the expression of TNF-α (*P*<0.05) and IL-6 (*P*<0.05) genes compared with the gentamicin group, however, the difference was not significant in the CAT gene (Figure 2). 

***Effect of cineole on renal tissue changes***

**Figure 3:**
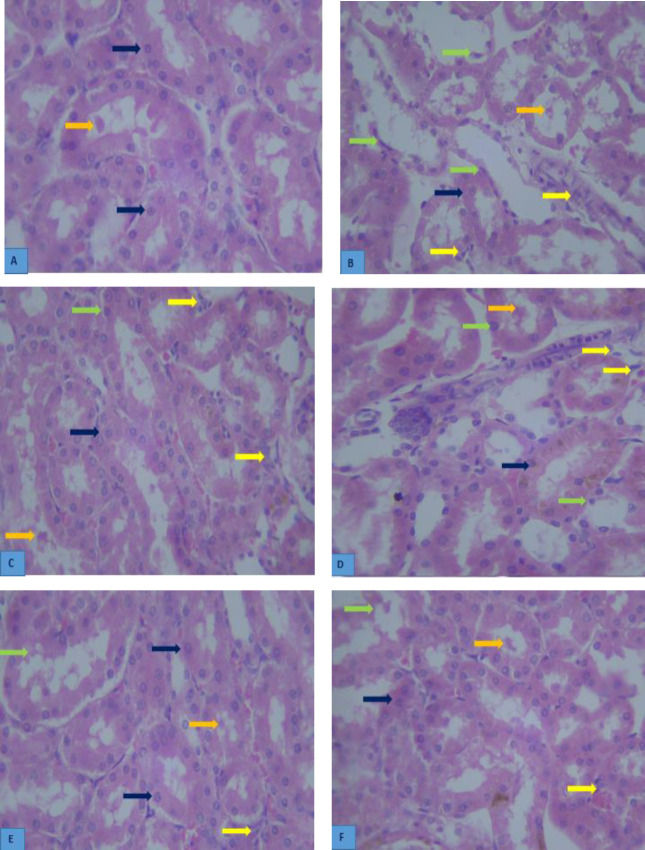
Effect of Cineole on histological changes of rat kidney induced by gentamicin in different groups. A: Control group, B: Gentamicin group, C: DMSO group D: Cineole 50 group, E: Cineole100 group, F: Vitamin E group, Photomicrographs (H&E, 400×). The black arrow shows the normal histological structure of renal tubular. The green arrow shows renal epithelial degeneration and tubular necrosis. The orange arrow shows the renal epithelial cast. The yellow arrow shows leukocytes infiltration in the interstitial area

Tubular necrosis, leukocyte infiltration, epithelial cast, and glomerular damage (atrophic glomeruli) of the renal tissue in the gentamicin group showed a considerable increase compared with the control group (*P*<0.05). Tubular necrosis, epithelial cast, and glomerular damage in the two cineole-receiving and vitamin E groups demonstrated a notable reduction compared with the gentamicin group (*P*<0.05). Leukocyte infiltration in the Cineole 100 group showed a significant decrease compared with the gentamicin group (*P*<0.05). Tubular necrosis, leukocyte infiltration, epithelial cast, and glomerular damage of the renal in the DMSO group showed a considerable decrease compared with the gentamicin group (*P*<0.05) (Table 4) (Figure 3). 

## Discussion

In the present study, gentamicin-induced nephrotoxicity caused an increase in serum urea, creatinine, levels of kidney MDA and serum NO, glomerular damage, and renal proximal convoluted tubules. The expression of TNF-α and IL-6 genes increased, and on the other hand, the levels of serum MDA, serum and kidney GSH and GPX, kidney CAT, and the expression of CAT and GPX genes decreased compared with the control group.

Cineole 50 and 100 groups increased the levels of serum GSH, kidney and serum GPX, kidney CAT, and serum NO compared with the gentamicin group. Moreover, these treatment groups decreased levels of kidney MDA, serum creatinine, and urine protein compared with the gentamicin group. Cineole 50 increased the serum MDA and serum urea and decreased the kidney GSH. Cineole 100 increased the kidney GSH and decreased the serum MDA and serum urea compared with the gentamicin group.

Previous studies have demonstrated that gentamicin reduces glomerular filtration rate, and decreased GFR appears to increase serum urea levels (28). Gentamicin causes lipid peroxidation by increasing reactive oxygen species, and on the other hand, it reduces the amount of antioxidants in renal tissue (29). Gentamicin causes oxidative stress and apoptosis by disrupting mitochondrial function in rat testis tissue, which as a result, reduces the testis weight and inhibits spermatogenesis (30). In addition, gentamicin increases the expression of inflammatory cytokines in renal tissue, thus, inflammation increases (31). Therefore, gentamicin increases oxidative stress and inflammation by increasing ROS and disrupting the balance of oxidants and antioxidants and leads to kidney function impairment. Polat *et al.* (2006) showed that NO synthesis increased following induction of nephrotoxicity by gentamicin (32), this study demonstrated that there were no remarkable differences in serum NO between experimental groups in general. In a study, it was shown that NO is effective in blocking acetaminophen-induced nephrotoxicity by reducing congestion and lipid peroxidation in mice (33). Moreover, NO causes dilation of blood vessels, relaxation of smooth muscles, nerve transmission, and immune response, and it can strengthen the oxidant system and eliminate free radicals, although it can be toxic because it is a free radical (34). On the other hand, cineole reduces the oxidative stress caused by nicotine by increasing the release of NO and inhibiting lipid peroxidation (35). Antioxidants are described as substances that are able to compete with other oxidants (compatible with oxygen) at relatively low concentrations and significantly inhibit or delay the oxidation of these substances (36, 37). Antioxidants such as rosmarinic acid (38), pomegranate (3, 36), camphor (12), and L-glutamine (39) can reduce the toxicity in different organs by reducing the amount of lipid peroxidation and increasing the activity of antioxidants such as GPX and CAT. In our study, the mean activity of CAT increased under the influence of cineole treatment in both 50 and 100 doses compared with the gentamicin group. Moreover, vitamin E increased the activity of the CAT enzyme in comparison with the gentamicin group. Serum GSH levels increased significantly in cineole treatment groups and kidney MDA decreased significantly in cineol and vitamin E groups compared with the gentamicin group. 

Cineole has anti-inflammatory properties that can be effective in curing cancers such as lung and liver cancers (40). Cineole leads to cell cycle arrest caused by ROS through regulating AMPK, Akt/mTOR, and MAPK pathways. Cineole with anticancer properties can be used as an adjuvant treatment with anti-neoplastic drugs in the treatment of cancers (40). Hence, we performed an experimental study to assess the effect of cineole, as an antioxidant, on the histology and change in renal function in gentamicin-induced nephrotoxicity in rats.

In the present study, Cineole and Vitamin E, as positive control, increased the expression of antioxidant genes. In a study, it was observed that cineole reduces ischemic stroke injuries by enhancing the activity of SOD and inhibiting the excessive production of ROS (41). Therefore, cineole by strengthening the antioxidant system destroys reactive oxygen species. 

Cineole has sedative, anti-anxiety, anti-depressant, and anti-insomnia activities that can be used to treat the post-traumatic stress disorder (42). Studies have shown that cineol is effective in treating inflammatory diseases such as bronchitis (43). Cineole has extensive medicinal properties, such as anti-inflammatory and antioxidant, which are mainly shown through the regulation of NF-kB and Nrf2, and is used to treat respiratory and cardiovascular diseases (44). Cineole improves diabetic nephropathy by reducing the formation of various glycation products, oxidative stress, and inflammatory pathways (45). Considering that cineole has an inhibitory effect on the function of circulating platelets, it can be effective in the prevention and treatment of thrombolytic disease (46). In a study, it was shown that cineole improved kidney tissue damage in animals with diabetic nephropathy (45) and in another study, the dose-dependent toxicity effect of cineole on liver and kidney tissue has been shown (47). Cineole is considered a suppressor for inflammatory responses due to its ability to reduce the levels of NO, TNF-α, and IL-6 expression (48). In our study, treatment with cineole and vitamin E caused a significant decrease in TNF-α and IL-6 gene expressions compared with the gentamicin group. Also, cineol with its antioxidant properties reduces oxidative stress in kidney tissue. The results of our study showed that cineole, with its antioxidant and anti-oxidative stress properties, reduced the injuries of the renal proximal convoluted tubule and glomerulus following gentamicin-induced nephrotoxicity. 

**Table 1:** Sequences of primers used in this study

**Product size** **(bp)**	**Sequence**	**Primer**	**Gene**
105	5´ATTGCCGTCCGATTCTCC3´	Forward	CAT
	5´CCAGTTACCATCTTCAGTGTAG3´	Reverse	
68	5´GGTGTTCCAGTGCGCAGAT3^´^	Forward	GPX
	5´AGGGCTTCTATATCGGGTTCGA3´	Reverse	
87	5´CCAGGAGAAAGTCAGCCTCCT3´	Forward	TNF-α
	5´ TCATACCAGGGCTTGAGCTCA3´	Reverse	
74	5´CGAAAGTCAACTCCATCTGCC3´	Forward	IL-6
	5´GGCAACTGGCTGGAAGTCTCT3´	Reverse	
**150**	**5´TATCGGCAATGAGCGGTTCC3´**	**Forward**	**β-actin**
**150**	**5´AGCACTGTGTTGGCATAGAGG3´**	**Reverse**	**β-actin**

**Table 2:** Mean activity of anti-oxidant enzymes, lipid peroxidation index, and tissue in experimental groups following gentamicin-induced nephrotoxicity and treatment with cineole and vitamin E in rats (n=6)

**Group**	mean ± SD
Control	^$^ 6.42±40
Gentamicin	14.46 * ±90.3
DMSO	17.35±71.16
50 mg/ kg Cineol	49.62 *±137.16
100 mg/ kg Cineol	13.04 *±65.66
VIT E	6.54 * ±96.4

Data were expressed as mean±SD. Data were analyzed by one-way ANOVA followed by Tukey’s *post hoc* test. * significant difference compared with the Control group (*P<*0.05), ^$^ significant difference compared with the gentamicin group (*P<*0.05)

**Table 3.** Serum urea levels in experimental groups following gentamicin-induced nephrotoxicity and treatment with cineole and vitamin E in rats (n=6)

Test	Tissue	Group						*P- *value
		Control	Gentamicin	DMSO	Cineole 50 mg/ kg	Cineole 100 mg/ kg	Vitamin E	
MDA µmol/mg protein	Kidney	3.6 ±45.72	.85 5±51.81	5.11^ $^ ±41.23	4.87^ *$^ ±33.44	7.51^ $^ ±37.06	5.54^ *$^ ±/ 32.26	<0.001
	Serum	0.20^ $^ ±1.78	0.23^ *^ ±1.60	0.02 ±/1.34	0.26^ *^ ±1.98	0.15 ±1.31	0.32 ±1.32	<0.001
GSH µmol/mg protein	Kidney	0.20 ±2.75	0.23 ±2.33	0.29 ±2.37	0.29 ^*^ ±2.21	1.16 ±2.38	0.0440±2.63	<0.05
	Serum	0.02 ±0.24	0.02 ±0.19	0.03 ±0.2	0.04 ^$^ ±0.27	0.04 ±0.23	0.04 ±0.23	<0.05
GPX Uni/mg protein	Kidney	10.83 ±254.49	23.37 ±229.49	20.57 ±251.90	58.53 ±305.71	37.52 ^$^ ±299.270	30.03 ^$^ ±298.32	<0.001
	Serum	4.99 ^$^ ±41.61	4.46 ^*^ ±32.49	4.23 ±38.11	9.12^ $^ ±51.75	7.08 ^$^ ±44.75	5.54^ $^ ±41.31	<0.001
Catalase Uni/mg protein	Kidney	0.7^ $^ ±5.84	0.38 ^*^ ±3.39	0.58 ^*^ ±3.8	0.15 ^$^ ±4.69	0.55 ^$^ ±4.5	1.38 ^$^ ±6.27	<0.001
NO µmol/mg protein	Serum	3.12^ $ ^±42.75	1.55 ^* ^±46.48	2.40^ $^ ±44.67	1.11^ *^ ±49.92	1.28^ *^ ±48.64	3.02^ *^ ±47.31	<0.001
Creatinin mg/dl	Serum	0.03^ $^ ±0.64	0.06^ *^ ±1.35	0.08 ^*$^ ±0.82	0.15^ *^ ±0.88	0.08^ $^ 0±0.75	0.13^ $^ ±0.71	<0.001

Data were expressed as mean±SD. Data were analyzed by one-way ANOVA followed by Tukey’s post hoc test. * significant difference compared with the Control group (*P<*0.05), ^$^ significant difference compared with the gentamicin group (*P<*0.05)

A: *significant difference (*P<*0.05); B: **significant difference (*P<*0.05); C: **significant difference compared with cineole 100 mg/kg group (*P<*0.05) and ***significant difference compared with gentamicin group (*P<*0.05); D: ***significant difference compared with gentamicin group (*P<*0.05)

**Table 4:** Histopathological changes of kidney tissue in experimental groups following gentamicin-induced nephrotoxicity and treatment with cineole and vitamin E in rats (n=6)

	Control	Gentamicin	DMSO	Cineol50	Cineol100	Vitamin E
Tubular necrosis	0.54±0.16^$^	1.58±0.23^*^	0.80±0.32^$^	0.88±0.14^$^	0.90±0.20^$^	0.73±0.06^$^
Leukocyte infiltration	0.3±0.08^$^	1.08±0.45^*^	0.57±0.13^$^	0.90±0.21^*^	0.70±0.08^*$^	1.01±0.14^*^
Eosinophilic casts	0.49±0.31^$^	1.25±0.54^*^	0.68±0.27^$^	0.67±0.24^$^	0.51±0.06^$^	0.44±0.03^*$^
Glomerular damage	0.23±0.09^$^	0.7±0.25^*^	0.45±0.06^*$^	0.44±0.04^*^^$^	0.43±0.03^*$^	0.46±0.05^*$^
						

Data were expressed as mean±SD. Data were analyzed by one-way ANOVA followed by Tukey’s *post hoc* test. * significant difference compared with Control group (*P<*0.05), ^$^significant difference compared with gentamicin group (*P<*0.05)

## Conclusion

The results of this research showed that cineole with antioxidant and anti-inflammatory properties decreased the expression of pro-inflammatory cytokines such as TNF-α and IL-6 and increased the expression of the GPX kidney antioxidant enzyme gene. Cineole reduced the damage to the glomerulus and curved tubes near the kidney, hence, it could improve kidney function by preserving kidney nephrons. However, more extensive research with more experimental groups including the investigation of apoptosis-related genes is needed for the beneficial effects of cineole in reducing gentamicin-induced nephrotoxicity.

## Authors’ Contributions

SRM, AV, LJ, and SV participated in data collection and drafting of the manuscript, data analysis, and interpretation of the results. HA, SRM, and AV conceived and designed the study. SRM, AV, LJ, and ZE wrote and revised the article. 

## Data availability statement

Upon reasonable request, the data supporting the results of this article will be available through the corresponding author.

## Conflicts of Interest

The authors declare that there are no conflicts of interest. 
